# α_1B_-Adrenergic Receptors Differentially Associate with Rab Proteins during Homologous and Heterologous Desensitization

**DOI:** 10.1371/journal.pone.0121165

**Published:** 2015-03-23

**Authors:** Jean A. Castillo-Badillo, Omar B. Sánchez-Reyes, Marco A. Alfonzo-Méndez, M. Teresa Romero-Ávila, Guadalupe Reyes-Cruz, J. Adolfo García-Sáinz

**Affiliations:** 1 Instituto de Fisiología Celular, Universidad Nacional Autónoma de México, Ap. Postal 70–248, México D.F. 04510, Mexico; 2 Departamento de Biología Celular, Centro de Investigación y de Estudios Avanzados del Instituto Politécnico Nacional-CINVESTAV, Av. Instituto Politécnico Nacional No. 2508, Col. San Pedro Zacatenco, CP 07360, México, D.F., Mexico; University of São Paulo, BRAZIL

## Abstract

Internalization of G protein-coupled receptors can be triggered by agonists or by other stimuli. The process begins within seconds of cell activation and contributes to receptor desensitization. The Rab GTPase family controls endocytosis, vesicular trafficking, and endosomal fusion. Among their remarkable properties is the differential distribution of its members on the surface of various organelles. In the endocytic pathway, Rab 5 controls traffic from the plasma membrane to early endosomes, whereas Rab 4 and Rab 11 regulate rapid and slow recycling from early endosomes to the plasma membrane, respectively. Moreover, Rab 7 and Rab 9 regulate the traffic from late endosomes to lysosomes and recycling to the trans-Golgi. We explore the possibility that α_1B_-adrenergic receptor internalization induced by agonists (homologous) and by unrelated stimuli (heterologous) could involve different Rab proteins. This possibility was explored by Fluorescence Resonance Energy Transfer (FRET) using cells coexpressing α_1B_-adrenergic receptors tagged with the red fluorescent protein, DsRed, and different Rab proteins tagged with the green fluorescent protein. It was observed that when α_1B_-adrenergic receptors were stimulated with noradrenaline, the receptors interacted with proteins present in early endosomes, such as the early endosomes antigen 1, Rab 5, Rab 4, and Rab 11 but not with late endosome markers, such as Rab 9 and Rab 7. In contrast, sphingosine 1-phosphate stimulation induced rapid and transient α_1B_-adrenergic receptor interaction of relatively small magnitude with Rab 5 and a more pronounced and sustained one with Rab 9; interaction was also observed with Rab 7. Moreover, the GTPase activity of the Rab proteins appears to be required because no FRET was observed when dominant-negative Rab mutants were employed. These data indicate that α_1B_-adrenergic receptors are directed to different endocytic vesicles depending on the desensitization type (homologous vs. heterologous).

## Introduction

The G protein-coupled receptor (GPCR) superfamily comprises more than 600 distinct seven transmembrane-spanning members, and represents the largest group of integral membrane proteins [1–4]. These receptors mediate a plethora of processes, from sexual reproduction in unicellular eukaryotes, such as yeasts, to olfaction, vision, cognition, pain perception, and endocrine and exocrine secretion in vertebrates, and they participate in essentially all major physiological processes in mammals. These receptors are key elements for organisms to sense the exterior and for tissues and cells to adjust to changes in the internal milieu. Not surprisingly, GPCRs are also involved in the pathogenesis of many diseases and are targets of a large percentage of prescribed and illegal drugs [1–4].

GPCRs do not remain static at the plasma membrane, but rather, they are continuously internalized and recycled back to the cell surface. In fact, internalization and recycling are central parts of the regulation of GPCR signaling. These processes take place under baseline conditions (i. e., in the absence of any stimulus and markedly modify their kinetics when cells are stimulated. Agonists induce internalization of their targeted GPCRs, which begins within seconds or minutes of exposure, contributing to receptor desensitization. Desensitization is an essential mechanism that prevents receptor overactivation, in response to intense or prolonged agonist stimulation; it is a conserved process for adjusting cell responsiveness (a classical example is the adjustment of rhodopsin to light intensities [5, 6]). Interestingly, a large amount of evidence indicates that the desensitization/ resensitization process should not be simply viewed as an off-on transition, but rather as signaling switches [7]. Current ideas indicate that signaling takes place not only at the plasma membrane but that GPCRs signal from endosomes [8]. Many GPCRs become reactivated (resensitized) following agonist removal and receptor dephosphorylation, a process that appears to require receptor endocytosis [9, 10]. The best defined endocytic route used by GPCRs is the clathrin-mediated pathway [11, 12].

The classical pathways of GPCR desensitization involve receptor phosphorylation by either second messenger-activated protein kinases or G-protein-coupled receptor kinases (GRKs), followed by β-arrestin binding and the formation of clathrin-coated vesicles [13]. β-Arrestins function as adapter/scaffolding proteins that allow GPCRs to incorporate into the formation of clathrin-coated vesicles through their association with both the β2-adaptin subunit of the AP2 adapter complex and clathrin itself. Once internalized, receptors are retained in early endosomes and either recycled back to the plasma membrane or marked for degradation in the lysosomes [14].

Endocytosis is characterized by vesicular transport along numerous pathways. In these processes, interactions of the receptor’s intracellular domains with specific targeting proteins appear to be crucial for sorting the internalized receptor among endosome types. Common steps in each pathway include membrane budding to form vesicles, transport to particular destinations and ultimately, docking and fusion with the target organelles, whose specificity is rendered in part by association with a key family of enzymes, the Rab GTPases [15, 16]. These small GTPases, which are monomeric G-proteins with molecular masses between 20 and 30 kDa [17], are regulatory proteins that participate in GPCR trafficking [18]. Activated Rab proteins selectively bind to effector proteins and can, in discrete steps, facilitate membrane transport. Rab proteins, temporally and spatially, coordinate vesicular transport through sequential interactions [15]. These proteins regulate vesicular transport, both in endocytosis and exocytosis, and have also been implicated in the control of vesicle docking and fusion [19–21]. There are more than 60 different Rab proteins, which comprise the largest family of monomeric small GTPases [16, 22]; the main characteristic of each Rab type is its distinct intracellular localization, likely associated with involvement in particular functions within the cell for regulating intracellular transport among organelles [16, 23, 24]. Initially, Rab proteins are recruited to and activated on the surface of membranes [25–27]; subsequently, they appear to facilitate vesicle transport along the cytoskeleton [28, 29], and finally, they participate in docking and fusion [19–21, 30, 31]. Thus, Rab proteins may be viewed as central regulators or integrators of vesicular transport.

Rab proteins, like other GTPases, cycle between an active state bound to GTP and an inactive state bound to GDP. These guanine nucleotide-bound states are regulated by the GTPase-activating proteins (GAPs), which accelerate the intrinsic rate of hydrolysis of bound GTP to GDP and by guanine nucleotide exchange factors (GEFs) that catalyze the exchange of bound GDP for GTP, or that inhibit GDP dissociation [16].

Accordingly, Rab 5 is associated with the plasma membrane, with clathrin-coated vesicles, and with early endosomes [17]. Other members of this family, i. e. Rab 4 and Rab 11, exhibit colocalization with Rab 5 in early endosomes. Rab 4 controls rapid recycling of proteins to the cell membrane [24] and the slow recycling of proteins take place through Rab 11. This latter Rab is located in early endosomes, perinuclear recycling endosomes and the trans-Golgi network and is considered to be the control of slow endosomal recycling and trafficking to the Golgi apparatus, and is also likely involved in trafficking to the plasma membrane [16, 24, 32, 33]. In addition, Rab 11 participates in GPCRs slow recycling [34]. Rab 7, another member of the Rab family, is located in late endosomes and lysosomes [35–37] and it has been suggested that it regulates the traffic from early to late endosomes and from there to lysosomes [18, 30].

After stimulation by agonist, several GPCRs traffic to late endosomes and lysosomes, with subsequent proteolytic degradation. α_1B_-Adrenergic receptors mediate many of the action of adrenaline and noradrenaline and thus, in their roles in health and disease [38, 39]. These receptors mainly exert their actions through coupling to Gq and phospholipase C which generate the two second messengers, inositol 1,4,5-trisphosphate and diacylglycerol, leading to calcium signaling and protein kinase C activation, respectively [38, 39]. α_1B_-Adrenergic receptor desensitization, phosphorylation, and internalization has been extensively studied and major differences have been observed in these processes when they are triggered either by adrenergic agonists (homologous) or by unrelated agents (heterologous) acting through other GPCRs, receptor tyrosine kinases, nuclear receptors or by direct activation of protein kinase C [40–57]. Among the GPCRs whose activation induce α_1B_-adrenergic receptor phosphorylation and internalization is the sphingosine 1-phosphate S1P1 receptor [42]. Sphingosine 1-phosphate is a bioactive lipid that functions as a paracrine / autocrine mediator [58], and through the S1P1 receptor plays cardinal roles in the intense crosstalk that takes place between receptor tyrosine kinases and GPCRs [42, 43, 58]. This sphingolipid induces very robust α_1B_-adrenergic receptor phosphorylation, desensitization and internalization through a signaling process involving phosphoinositide 3-kinase and protein kinase C activities [42, 59].

As mentioned previously, regulation of GPCR vesicular trafficking involves the activity of different Rab proteins. On this basis, we hypothesized that differences might exist in their involvement in internalization associated with homologous and heterologous desensitization. Here we show that when α_1B_-adrenergic receptors are desensitized by noradrenaline (homologous) these adrenergic receptors interact with Rab proteins associated with early endosomes and fast recycling, such as Rab 5 and Rab 4, whereas when the adrenergic receptor is desensitized by the action of sphingosine 1-phosphate (heterologous) it rapidly but transiently interacts with Rab 5 and subsequently with proteins from late endosomes, such as Rab 7 and Rab 9.

## Materials and Methods

### 1. Materials

Sphingosine 1-phosphate, dl-propranolol, (-)-noradrenaline and DNA purification kits were purchased from Sigma Chemical Co. Dulbecco’s modified Eagle’s medium, wheat germ agglutinin-Alexa Fluo 350, fetal bovine serum, trypsin, antibiotics and other reagents used for cell culture were from Life Technologies. Fura 2AM was obtained from Invitrogen and agarose-coupled protein A from Upstate Biotechnology. Rabbit antisera against the enhanced green fluorescent protein (EGFP) and the α_1B_-adrenergic receptor carboxyl terminus decapeptide were generated in our laboratory and have been previously characterized [42, 57, 60, 61]. A rabbit antiserum against the DsRed fluorescent protein was also obtained in our laboratory. A plasmid for expression of DsRed in *Escherichia coli* was generously provided to us by Dr. Stefan Jakobs (Max Planck Institute for Biophysical Chemistry, Goettingen, Germany) [62]. The protein was expressed and purified as described [62] and rabbits were immunized. The resulting antiserum was suitable for Western blot analysis and immunoprecipitation (vide infra). For the immunostaining studies a commercial, purified goat polyclonal antibody was obtained from Santa Cruz Biotechnology and an Alexa Fluor 594-conjugated affinity-purified Donkey anti-goat IgG was used (Jackson ImmunoResearch), other secondary antibodies were obtained from Zymed. The vectors, pEGFP-N1 and pDsRed-Monomer-N1, were obtained from Clontech. The human α_1B_-adrenergic receptor coding sequence inserted into the pEGFP-N1 vector [48] was excised (BgIII—EcoRI), purified and subcloned into pDsRed-Monomer-N1 (NheI—EcoRI) in order to tag it with the red fluorescent protein, DsRed. Proper insertion was confirmed by restriction analysis and sequencing at the Molecular Biology Unit of our Institute. EGFP-tagged wild-type and mutant Rab GTPases were generated by Dr. Robert Lodge (Institut de Recherches Cliniques de Montréal, Montreal, Canada) [63] and generously provided to us. The plasmid pCEFL-EGFP β-arrestin2 CAAX for expression of a membrane-directed EGFP-tagged β-arrestin 2 mutant was kindly provided to us by Dr. Silvio Gutkind (National Institutes of Health; Bethesda, MD, USA) [64].

### 2. Cell lines

A cell line stably expressing human α_1B_-adrenergic receptors tagged with the red fluorescent protein (DsRed) was generated as follows: HEK293 AD cells (American Type Culture Collection) were cultured in Dulbecco’s modified Eagle’s medium containing glutamine and high-glucose and was supplemented with 10% fetal bovine serum, 100 μg/ml streptomycin, 100 U/ml penicillin, and 0.25 μg/ml amphotericin B at 37°C under a 95% air/5% CO_2_ atmosphere. Cells were transfected with lipofectamine 2000 following the supplier’s instructions using the plasmid described previously. After 24 h, the medium was supplemented with 300 μg/ml of the neomycin analog, G-418 sulfate, for selection, and was maintained in this medium throughout the course of all of the experiments. A cell line was selected on the basis of the expression of the adrenergic receptor tagged with DsRed as evidenced by the following: a) epifluorescence microscopy, b) confocal fluorescence microscopy and c) the ability of noradrenaline to increase the intracellular calcium concentration. In all experiments employing noradrenaline, 1 μM propranolol was present to block endogenous β-adrenergic receptors.

### 3. Intracellular calcium determinations and α_1B_-adrenoceptor phosphorylation

Calcium determinations were performed as described [42]. In brief, cells were loaded with 2.5 μM Fura-2/AM and washed to eliminate unincorporated dye. Fluorescence measurements were carried at 340 and 380 nm excitation wavelengths and 510 nm emission wavelength, with a chopper interval set at 0.5 sec using an AMINCO-Bowman Series 2 luminescence spectrometer (Rochester, NY, USA). Intracellular calcium ([Ca^2+^]i) was calculated according to Grynkiewicz et. al. [65].

The procedure employed to study α_1B_-AR phosphorylation, has been previously described in detail [42, 48, 57, 66]. In brief, cells were maintained overnight in phosphate-free Dulbecco’s modified Eagle’s medium without serum. The following day, cells were incubated in 1 ml of the same medium containing [^32^P]Pi (50 μCi/ml) for 3 h at 37°C. Labeled cells were stimulated as indicated (times for maximal effects were selected), washed, and solubilized. Cell lysates were centrifuged at 12,700 x *g* for 15 min at 4°C and supernatants were incubated overnight at 4°C with antisera and protein A-Sepharose. After two washes, pellets containing the immune complexes were boiled for 5 min in SDS-sample buffer containing 5% β-mercaptoethanol, and subjected to SDS-polyacrylamide gel electrophoresis. Gels were dried and exposed for 18–24 h and level of receptor phosphorylation was assessed with a Molecular Dynamics PhosphorImager using the ImageQuant software (Amersham Biosciences). Data fell within the linear range of detection of the apparatus and were plotted using Prism 5 software.

### 4. Transient transfection of proteins tagged with EGFP

To study the interaction between α_1B_-adrenergic receptors with β-arrestin, the different Rab proteins or early endosomes antigen 1 (EEA1), the cell line expressing the DsRed-tagged adrenoceptor was transfected with the corresponding plasmids utilizing Lipofectamine 2000, following the manufacturer’s instructions and cultured as described previously. Experiments were carried out 72 h post-transfection.

### 5. Western blot and coimmunoprecipitaton assays

Cells at 90–100% confluence were washed with ice-cold phosphate-buffered saline and lysed on ice for 1 h in buffer containing NaCl 150 mM, Tris 10 mM (pH 7.4), sodium cholate 1%, EDTA 5 mM, 1% Nonidet P40 and protease and phosphatase inhibitors. Lysates were centrifuged at 12,700 x g for 15 min and the whole cell lysate was incubated overnight with protein A-agarose and anti-EGFP antibody at 4°C. Protein A-agarose immunocomplexes were washed three times with lysis buffer, and the immunoreactive proteins bound on agarose were eluted in sample buffer containing dithiothreitol 0.1 M. Proteins were separated using 10% SDS-PAGE. Proteins were electrotransferred onto nitrocellulose membranes and immunoblottings were performed. Incubation with the primary selective antibodies was conducted for 12 h at 4°C and with the secondary antibody, for 1 h at room temperature. Super signal-enhanced chemiluminescence kits were employed exposing the membranes to X-Omat X-ray films. Signals were quantified by densitometric analysis utilizing the ImageJ software [67].

### 6. Confocal microscopy

To study β-arrestin-α_1B_-adrenergic receptor interaction, cells expressing DsRed-tagged adrenoceptors were transfected with a plasmid expressing a membrane-directed EGFP-tagged β-arrestin 2 [64] and intracellular colocalization was studied with an Olympus confocal microscope (vide infra). Membrane was delineated with the aid of the plasma membrane marker, wheat germ agglutinin-Alexa 350, and fluorescence was eliminated employing the ImageJ software [67], to define internalization and colocalization.

To study vesicular trafficking of α_1B_-adrenergic receptors and their interaction with different Rab proteins, cells stably transfected with human α_1B_-adrenergic receptors tagged with DsRed were further transfected with the indicated EGFP-tagged wild type or mutant Rab GTPases. Interaction between the receptor and Rab proteins was analyzed using FRET by the sensitized-emission method employing a confocal microscope equipped with an automated laser spectral scan FV10i Olympus. Briefly, cells were cultured in glass-bottomed Petri dishes for 24 h and treated with 1 μM sphingosine 1-phosphate or 10 μM noradrenaline (plus 1 μM propranolol) during 15 min and immediately fixed with 4% paraformaldehyde. Images of at least 5 different cell preparations were obtained utilizing a Fluoview Confocal microscope FV10i (Olympus, LD laser, 405 nm [18 mW], 473nm [12.5 mW], 635 nm [10 mW], 559nm) with a water-immersion objective (60X). EGFP was excited at 480 nm and the emitted fluorescence was detected at 515–540 nm, the red fluorescent protein, DsRed, was excited at 557 nm and the fluorescence emitted was detected at 592 nm. At least 15 images per sample were captured to estimate the interaction of Rab proteins with the adrenergic receptor by the FRET index after treatment with either agent. The FRET index was quantified using ImageJ software and the "FRET and Colocalization Analyzer" plugin [67].

For the immunostaining studies cells were cultured in glass-bottomed Petri dishes with glass bottom for 48 h and treated with 1 μM sphingosine 1-phosphate or 10 μM noradrenaline (plus 1 μM propranolol) during 15 min and immediately fixed with 4% paraformaldehyde and permeabilized with triton 0.1% and blocked with horse serum (10%) and bovine serum albumin (5%.). The preparations were incubated overnight with a 1:100 dilution of the commercial anti-α_1B_-adrenergic receptor antibody, and then washed 3 times and then incubated with the secondary Alexa-594 conjugated antibody. Samples were observed and imaged obtained using an Olympus Fluoview Confocal microscope FV10i.

### 7. Image analysis using ImageJ software

ImageJ version 1.47b was obtained from the National Institutes of Health website (ImageJ, Rasband, W.S., U. S. National Institutes of Health, Bethesda, MD, http://imagej.nih.gov/ij/). Images were processed according to the user guide of the "FRET and colocalization analyzer” plugin of the ImageJ software. This plug-in works with 8-bit images [67] and allows supervised computation of the FRET index by means of a “pixel by pixel” method. It requires sets of images to be acquired according to FRET computation by sensitized emission of the fluorescence method. During analysis with this plugin, controls are displayed to the user in order to confirm the results: among these are the following: a) Bleed Through Control (shows artifacts that could lead to a false FRET); b) FRET calculation min and max control (shows intervals of confidence of FRET index calculations); and c) FRET and colocalization diagram control (allow users to associate FRET with colocalization information, thus eliminating false FRET).

### 8. Spectrofluorometric FRET Analysis

The FRET assay and intensities were recorded using an AMINCO-Bowman Series 2 luminescence spectrometer (Rochester, NY, USA). Cells were loaded in Krebs-Ringer-HEPES containing 0.05% bovine serum albumin, pH 7.4 for 1 h at 37°C and then washed one time to eliminate the medium. The EGFP was excited at 488 nm, and fluorescence emission assay was captured from 490 to 600 nm by shifting the emission monochromator back and forth. Excitation and emission slits were set to 1 and 8 nm, respectively. Sample temperature was regulated by a circulating water bath. The emission spectrum was captured at time zero (baseline) and then 15 minutes after the stimulus, using emission scan.

### 9. Statistical Analysis

Statistical analysis between comparable groups was performed using ANOVA with Bonferroni’s post-test and was performed with the software included in the GraphPad Prism program.

## Results

**Fig 1:**
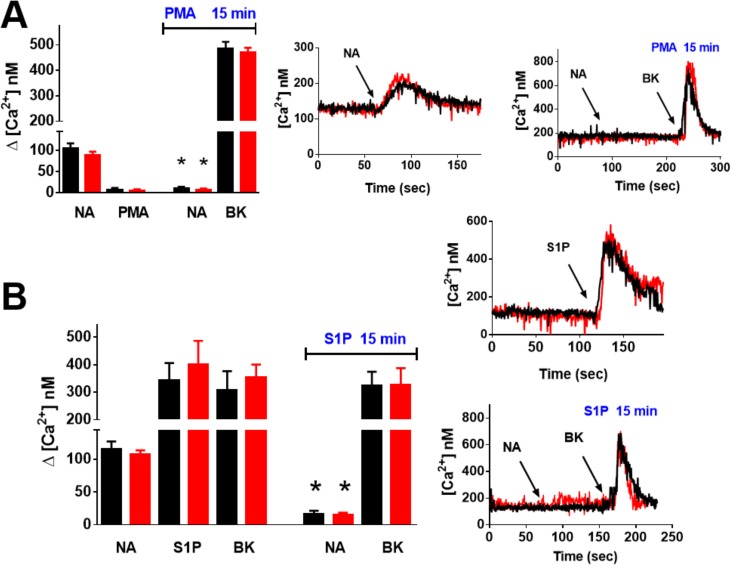
α_1B_-Adrenergic receptor action and desensitization. Panel A: Cells expressing wild-type (black symbols) or DsRed-tagged (red symbols) human α_1B_-adrenergic receptors were pre-incubated for 15 min in the absence or presence or 1 μM phorbol myristate acetate (PMA) and then were challenged with 10 noradrenaline (NA, arrow), 1 μM PMA or 1 μM bradykinin (BK) and intracellular calcium concentration was recorded. Plotted in the left figure are the means and vertical lines that represent the S.E.M. of 4–6 experiments using different cell preparations. *p < 0.001 vs. NA action in cells pre-incubated without PMA. Middle and right figures are representative tracings. Panel B: Cells expressing wild-type (black symbols) or DsRed-tagged (red symbols) human α_1B_-adrenergic receptors were pre-incubated for 15 min in the absence or presence or 1 μM sphingosine 1-phosphate (S1P) and then were challenged with 10 noradrenaline (NA, arrow), 1 μM S1P or 1 μM bradykinin (BK) and intracellular calcium concentration was recorded. In the left figure the means and vertical lines are plotted, that represent he S.E.M. of 4–6 experiments using different cell preparations. *p < 0.001 vs. NA action in cells pre-incubated without S1P. Middle and right figures are representative tracings.

**Fig 2:**
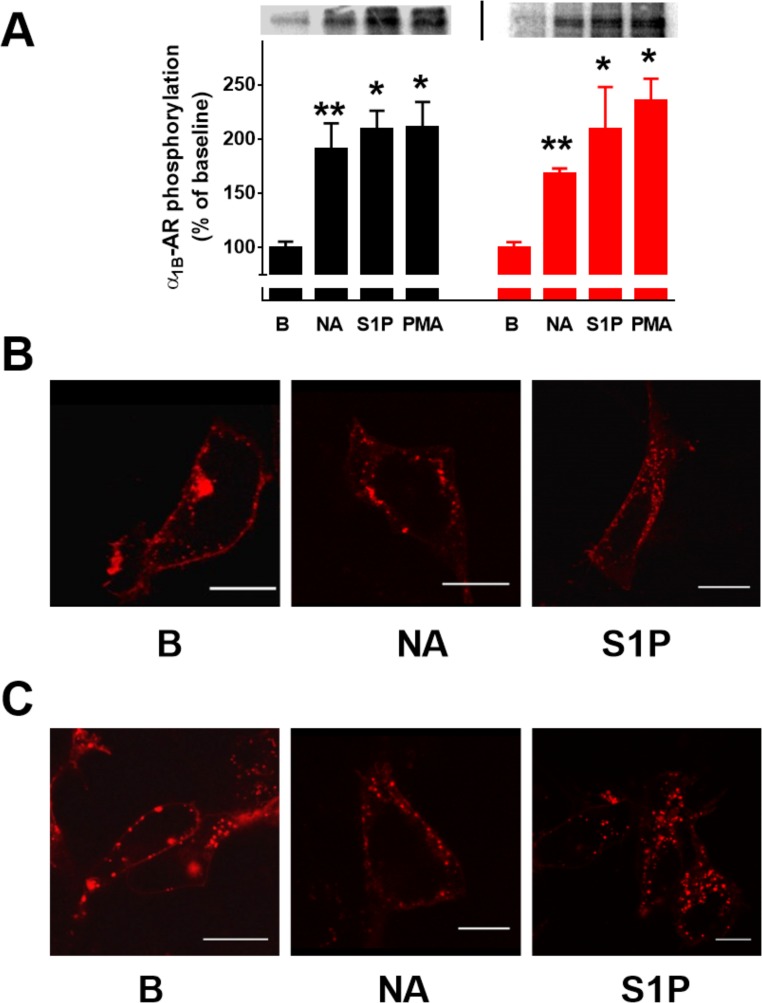
α_1B_-Adrenergic receptor phosphorylation and internalization. Panel A: Cells expressing wild-type (black symbols) or DsRed-tagged (red symbols) human α_1B_-adrenergic receptors were incubated in the absence of any agent (B, baseline, 30 min), 10 μM noradrenaline (NA, 15 min) or 1 μM sphingosine 1-phosphate (S1P, 30 min) to study receptor phosphorylation state. Plotted are the means and vertical lines representing the S.E.M. of 3–4 experiments using different cell preparations. *p < 0.005 vs. B (their respective cell line baseline value), **p < 0.05 vs. B (their respective baseline value). Representative autoradiographs are presented. Panel B: Confocal microscopy images of cells expressing DsRed α_1B_-adrenergic receptors incubated for 15 min in the absence of any agent (B, baseline), 10 μM noradrenaline (NA) or 1 μM sphingosine 1-phosphate (S1P). Panel C: Confocal microscopy images of cells expressing wild-type α_1B_-adrenergic receptors incubated for 15 min in the absence of any agent (B, baseline), 10 μM noradrenaline (NA) or 1 μM sphingosine 1-phosphate (S1P). Scale bars: 15 μm.

Noradrenaline was unable to increase intracellular calcium concentration in untransfected HEK293 cells but induced an immediate and robust response, in cells expressing human wild type or DsRed-tagged α_1B_-adrenergic receptors (Fig. 1, panel A). As expected, in these cells, activation of protein kinase C with phorbol myristate acetate essentially abolished the effect of noradrenaline on this parameter, without altering baseline intracellular calcium or bradykinin action (Fig. 1, panel A). Sphingosine 1-phosphate induced an immediate, robust increase in intracellular calcium concentration (Fig. 1, panel B). When cells were incubated with this bioactive lipid for 15 min, the effect of noradrenaline was markedly desensitized but the action of bradykinin was not altered (Fig. 1, panel B). These data indicate that sphingosine 1-phosphate induced heterologous α_1B_-adrenergic desensitization and that the effect is not due to intracellular calcium depletion. The data were essentially identical when the wild type or DsRed-tagged receptors were used and are entirely consistent with data obtained using EGFP-tagged α_1B_-adrenergic receptors [42]. Similarly, we observed that wild type and DsRed-tagged α_1B_-adrenergic receptors are phosphoproteins whose phosphorylation state is increased by agents that induce desensitization such as noradrenaline (homologous) or sphingosine 1-phosphate (heterologous) (Fig. 2, panel A). Internalization of Wild-type and DsRed-tagged α_1B_-adrenergic receptors after 15 min stimulation with noradrenaline (10 μM) or sphingosine 1-phophate is depicted in Fig. 2 (panels B and C, respectively). Internalization was mainly evidenced by a decrease in the delineation of the plasma membrane by fluorescence and by an increase in the number and intensity of fluorescent vesicles, which is consistent with what has been observed with EGFP-tagged α_1B_-adrenergic receptors [42].

**Fig 3:**
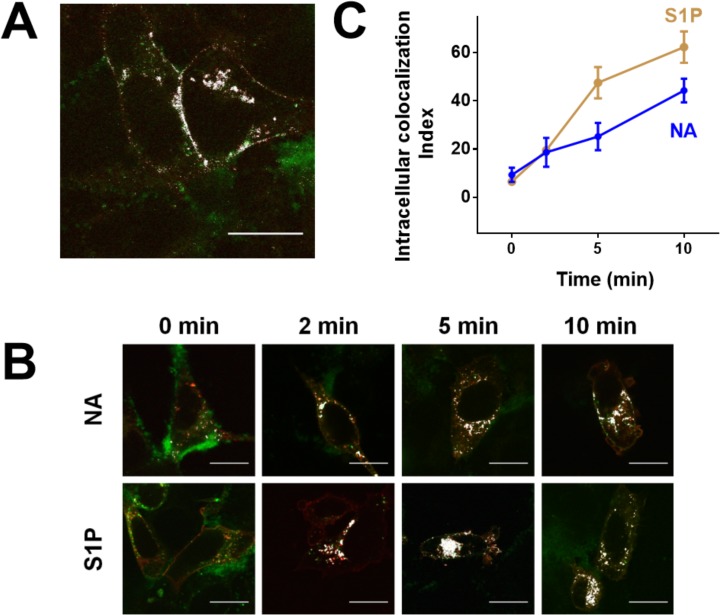
DsRed-tagged α1B-adrenergic receptor and membrane-directed EGFP-tagged β-arrestin 2 colocalization. Panel A: Representative image of cells expressing DsRed-tagged human α1B-adrenergic receptors and membrane-directed EGFP-tagged β-arrestin 2; colocalization is indicated in white. Panel B: Cells were incubated for the times indicated in the presence of 10 μM noradrenaline (NA) or 1 μM sphingosine 1-phosphate (S1P) and images were obtained. Plasma membrane fluorescence was deleted and intracellular colocalization is indicated in white. Panel C: Images obtained as indicated in Panel B were analyzed for intracellular colocalization of adrenergic receptors and β-arrestin. Plotted are the means and vertical lines representing the S.E.M. of 3–4 experiments using different cell preparations and 4–5 images were obtained and analyzed, from each condition. Scale bars: 15 μm.

Receptor interaction with β-arrestins appears to be a very initial step in clathrin-coated pit formation and internalization of GPCRs. Therefore, we explored the possibility that these proteins could internalize with DsRed-tagged α_1B_-adrenergic receptors during homologous and heterologous desensitizations. For this purpose a membrane-directed EGFP-tagged β-arrestin 2 mutant was transfected in the cell line expressing DsRed-tagged α_1B_-adrenergic receptors. As illustrated in Fig. 3 (panel A), β-arrestins and the adrenoceptors colocalize (white spots) mainly at the plasma membrane and to a lesser extent in intracellular vesicles. We next studied the effect of noradrenaline and sphingosine 1-phosphate on internalization/ colocalization of these proteins. Colocalization at the plasma membrane was eliminated from the images, guided with the use of the lectin plasma membrane marker, wheat germ agglutinin-Alexa 350, with the aid of ImageJ software. As presented in Fig. 3 (panel B), the data show that cell incubation with noradrenaline or sphingosine 1-phosphate induced receptor and beta-arrestin internalization that colocalize. These effects were time-dependent as evidenced by colocalization quantitation (Fig. 3, panel C).

The possibility that α_1B_-adrenergic receptors could directly interact with different Rab proteins and endosomal markers was explored by using the sensitized emission FRET, one of the most commonly employed methods to study FRET, based on image processing [67, 68]. Hence, we first generated a stable cell line expressing α_1B_-adrenergic receptors tagged with the DsRed fluorescent protein, which worked as an acceptor and transfected the cells with the different Rab proteins or the early endosomal marker tagged with EGFP, which could serve, when externally excited, as donors in FRET experiments. The excitation and emission characteristics of these fluorescent proteins render them a very suitable pair for these studies [69]. It is important to bear in mind that the FRET phenomenon is produced, if and only if, the distance between the fluorescent proteins is 10 nm (100 Å) or less [70], indicating close proximity, which suggests the possibility of a direct molecular interaction; this latter point needs to be addressed using structural approaches.

**Fig 4:**
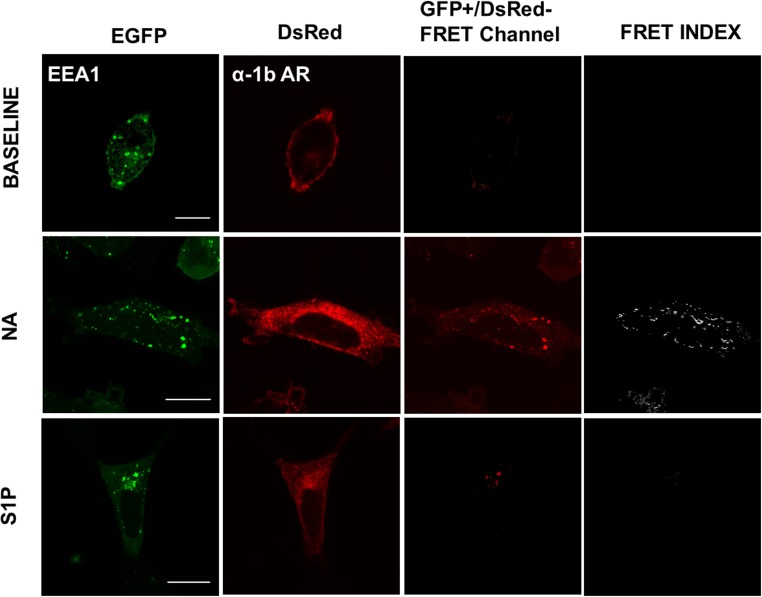
α_1B_-Adrenergic receptor-early endosome antigen 1 interaction. Images of cells coexpressing α_1B_-adrenergic receptors tagged with the DsRed fluorescent protein (α_1B_-AR) and the early endosomes antigen 1 (EEA1) tagged with the enhanced green fluorescent protein (EGFP). Cells were incubated for 15 min in the absence of any agent (B, upper row) or presence of 10 μM noradrenaline (plus 1 μM propranolol) (NA, middle row) or 1 μM sphingosine 1-phosphate (S1P, lower row). Cells were fixed and observed in a fluorescence confocal microscope. The following images are presented: EGFP fluorescence (EGFP was excited and its fluorescence recorded; first column), DsRed fluorescence (DsRed was excited and its fluorescence recorded; second column), “FRET channel” (EGFP was excited, the laser to excite DsRed remained off, and DsRed fluorescence was recorded; third column) and “FRET index” (images processed with the "FRET and Colocalization Analizer", fourth column). Scale bars: 15 μm.

**Fig 5:**
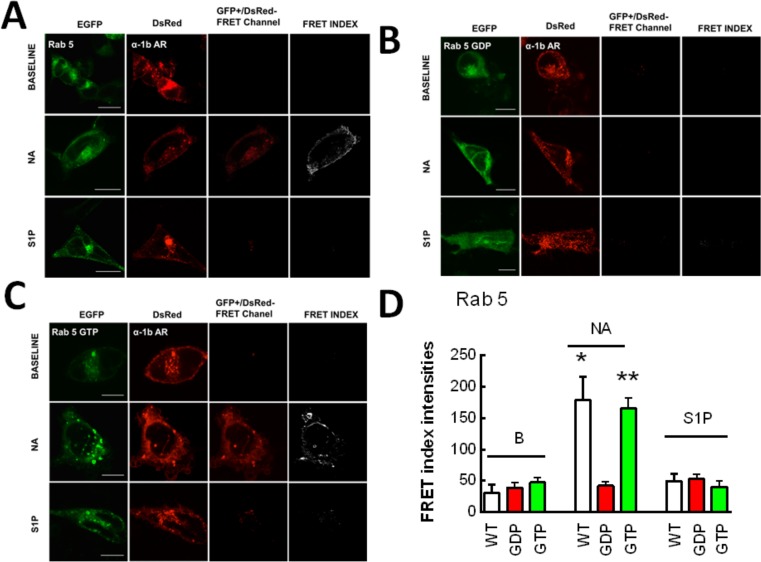
α_1B_-Adrenergic receptor-Rab 5 interaction. Images of cells coexpressing α_1B_-adrenergic receptors tagged with the DsRed fluorescent protein (α_1B_-AR) and Rab 5 tagged with the enhanced green fluorescent protein (EGFP). Other indications as in Fig. 1. Panel A, wild-type Rab 5 (WT); panel B, dominant-negative Rab 5 (Rab 5-GDP); and panel C, constitutively active Rab 5 (Rab 5-GTP). In panel D, the quantitative analysis of the FRET index is presented. Plotted are the means and vertical lines representing the S.E.M of 5–7 experiments using different cell preparations. * p< 0.001 vs. wild-type baseline (B) and stimulated with sphingosine 1-phosphate (S1P). ** p< 0.001 vs. Rab 5-GTP baseline (B) and stimulated with sphingosine 1-phosphate (S1P). Scale bars: 15 μm.

**Fig 6:**
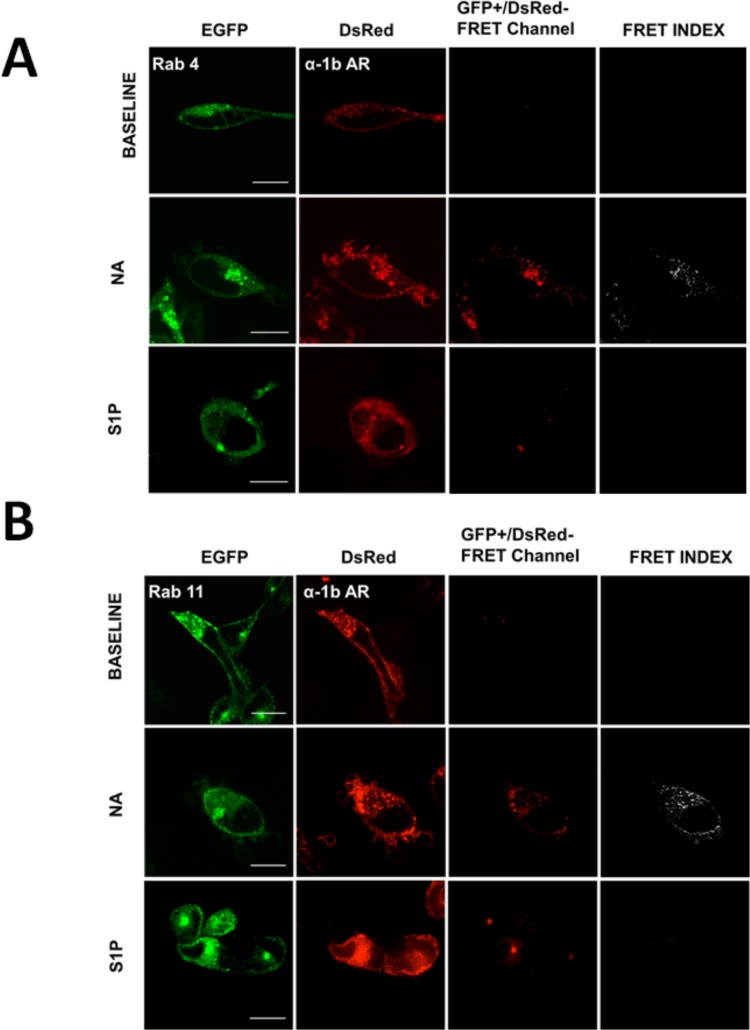
α_1B_-Adrenergic receptor-Rab 4 and Rab 11 interactions. Images of cells coexpressing α_1B_-adrenergic receptors tagged with the DsRed fluorescent protein (α_1B_-AR) and Rab 4 (panel A) or Rab 11 (panel B) tagged with the enhanced green fluorescent protein (EGFP). Scale bars: 15 μm.Other indications as in Fig. 1.

**Fig 7:**
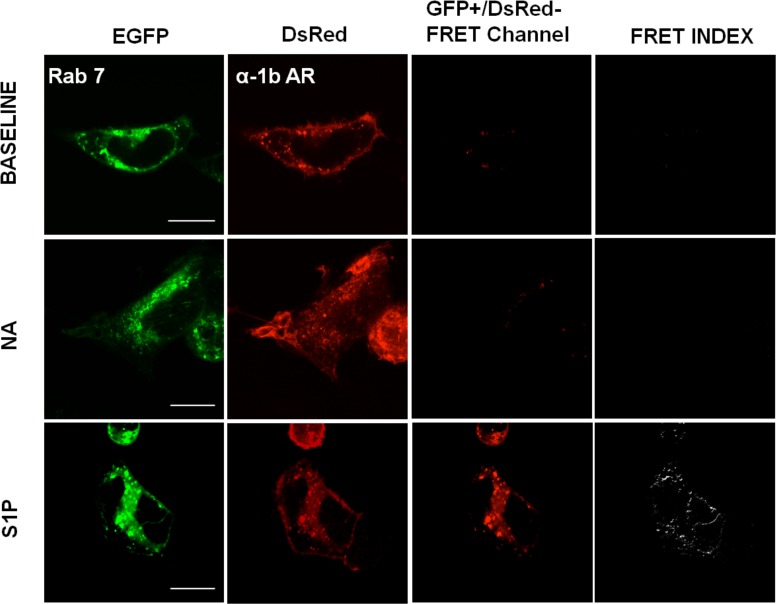
α_1B_-Adrenergic receptor-Rab 7 interaction. Images of cells coexpressing α_1B_-adrenergic receptors tagged with the DsRed fluorescent protein (α_1B_-AR) and Rab 7 tagged with the enhanced green fluorescent protein (EGFP). Scale bars: 15 μm. Other indications as in Fig. 1.

**Fig 8:**
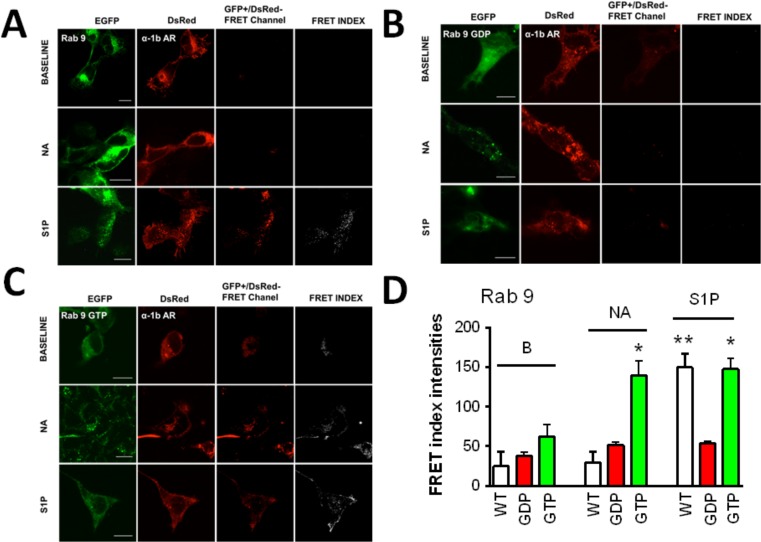
α_1B_-Adrenergic receptor-Rab 9 interaction. Images of cells coexpressing α_1B_-adrenergic receptors tagged with the DsRed fluorescent protein (α_1B_-AR) and Rab 9 tagged with the enhanced green fluorescent protein (EGFP). Other indications as in Fig. 1. Panel A, wild-type Rab 9 (WT); panel B, dominant-negative Rab 9 (Rab 9-GDP); and panel C, constitutively-active Rab 9 (Rab 9-GTP). In panel D, the quantitative analysis of the FRET index is presented. Plotted are the means and vertical lines representing the S.E.M of 5–10 experiments using different cell preparations. * p< 0.001 vs. Rab 9-GTP baseline (B); ** p< 0.001 vs. wild-type baseline (B) and wild-type stimulated with noradrenaline (NA). Scale bars: 15 μm.

In Figs. 4–8 the following groups of images are presented: EGFP fluorescence (EGFP was excited and its fluorescence recorded; first column), DsRed fluorescence (DsRed was excited and its fluorescence recorded; second column), and “FRET channel” (EGFP was excited, the laser to excite DsRed remained off, and DsRed fluorescence was recorded; third column). Images were processed with ImageJ software using the "FRET and Colocalization Analyzer" plugin [67] and the FRET index images (fourth column) are presented. Cells were incubated for 15 min with no agent (B, upper row), 10 μM noradrenaline (plus propranolol) (NA, middle row) or 1 μM sphingosine 1-phosphate (S1P, lower row). Propranolol by itself induced no effect on these parameters (data not shown). Concentration of agents and time of exposure were defined in preliminary experiments using continuous recordings in the “FRET channel”.

**Fig 9:**
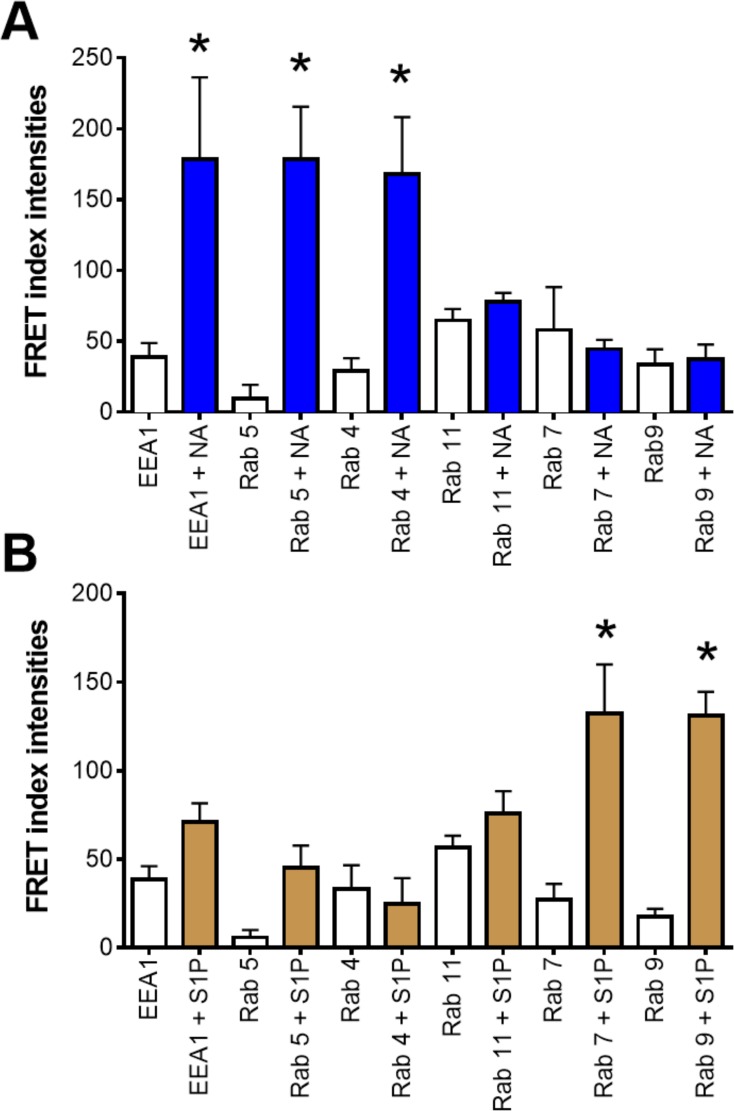
FRET index quantification. Panel A: plotted are the data obtained from cells incubated for 15 min in the absence of any agent (open bars) or with 10 μM noradrenaline (plus 1 μM propranolol)(NA, blue bars). Panel B: plotted are the data obtained from cells incubated for 15 min in the absence of any agent (open bars) or with 1 μM sphingosine 1-phosphate (S1P, brown bars). In the abscissa the protein tagged with EGFP is indicated. Plotted are the means with vertical lines representing the S.E.M. of 5 to 7 determinations using different cell preparations. *p< 0.05 vs. absence of stimuli.

Fig. 4 shows images obtained with cells expressing early endosomes antigen 1 (EEA1) (EGFP, first column) and α_1B_-adrenergic receptors (DsRed, second column). Early endosome antigen I fluorescence was present in the whole cytoplasm and enriched in different cell locations in a punctuated form (Fig. 4, first column). Similar patterns of cells distribution were observed with the Rab proteins (Figs. 5–8). Fluorescence due to the α_1B_-adrenergic receptor-DsRed construction was clearly evidenced in delineating of the plasma membrane and also in intracellular vesicles (Fig. 4, second column and subsequent figures, same column). No significant fluorescence was detected, for any of the proteins, within the nuclear region of the cells. As shown in Fig. 4 (third column), no FRET was observed under baseline conditions, but it was very clear in cells stimulated during 15 min with 10 μM of noradrenaline. Interestingly, no significant FRET was observed in cells stimulated with 1 μM sphingosine 1-phosphate. The FRET index images are presented in Fig. 4 (fourth column), which depicts essentially the same pattern as the FRET channel, but in a clearer and more reliable manner (due to the elimination of fluorescence that does not pass the “pixel by pixel” test). Quantitative analysis of FRET indexes for the EEA1 and wild-type Rab proteins images is presented in Fig. 9 (panel A, noradrenaline (NA); panel B, sphingosine 1-phosphate (S1P)).

Similar experiments were performed in cells expressing α_1B_-adrenergic receptors and the different Rab proteins, and these are illustrated in Figs. 5–8. It can be observed that noradrenaline clearly induced FRET in cells expressing Rab 5 (Fig. 5, panel A), Rab 4 (Fig. 6, panel A), and Rab 11 (Fig. 6, panel B) but not in cells expressing Rab 7 (Fig. 7) or Rab 9 (Fig. 8, panel A). The FRET index analysis presented in Fig. 9 (panel A) illustrates that there is a significant difference only with EEA1, Rab 5 and Rab 4.

When the cells were stimulated with sphingosine 1-phosphate a very different pattern was observed, i. e., cell treatment with the sphingolipid increased FRET in cells expressing Rab 7 (Fig. 7) or Rab 9 (Fig. 8, panel A) but not in those expressing Rab 5 (Fig. 5), Rab 4 (Fig. 6, panel A) or Rab 11 (Fig. 6, panel B). Fig. 9 (panel B), shows the FRET index analysis; the action of sphingosine 1-phosphate was statistically significant only with Rab 7 and Rab 9.

To further substantiate these findings, experiments were performed in cells expressing dominant-negative or constitutively active Rab proteins tagged with EGFP. Rab 5 and Rab 9 were selected (for early and late endosomes, respectively), on the basis of the data shown and the information available on their roles.

When the dominant negative mutant of Rab 5 (Rab 5-GDP) was employed we were unable to detect FRET when cells were stimulated by either noradrenaline or sphingosine 1-phosphate (Fig. 5, panel B). In contrast, very clear FRET was observed in response to noradrenaline, in cells expressing the constitutively active mutant of Rab 5 (Rab 5-GTP) (Fig. 5, panel C); no significant FRET was observed in these cells when they were stimulated with spingosine 1-phosphate (Fig. 5, panel C). The FRET index analysis presented in Fig. 5 (panel D) demonstrated that the action of noradrenaline was statistically significant.

**Fig 10:**
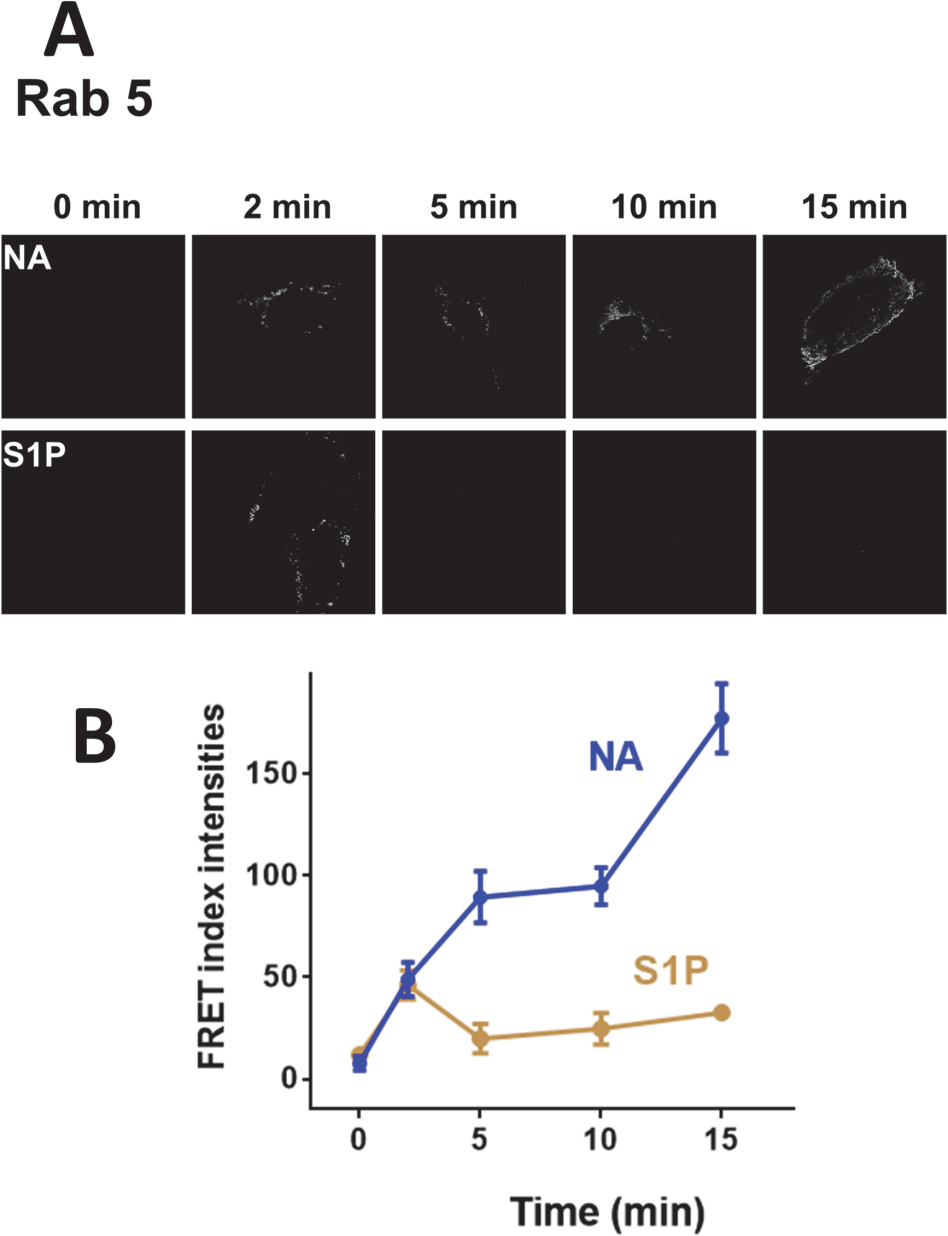
Time-course of the α_1B_-adrenergic receptor-Rab 5 interaction. Panel A: FRET index images of cells coexpressing Ds-Red-tagged α_1B_-adrenergic receptors and EGFP-tagged Rab 5 treated for the times indicated with 10 μM noradrenaline (NA) or sphingosine 1-phosphate (S1P). Scale bars: 15 μm. Panel B: Quantitative analysis of the data presented in Panel A. Plotted are the means and vertical lines representing the S.E.M of 5–7 experiments using different cell preparations. Noradrenaline, blue line and symbols; sphingosine 1-phosphate, brown line and symbols.

**Fig 11:**
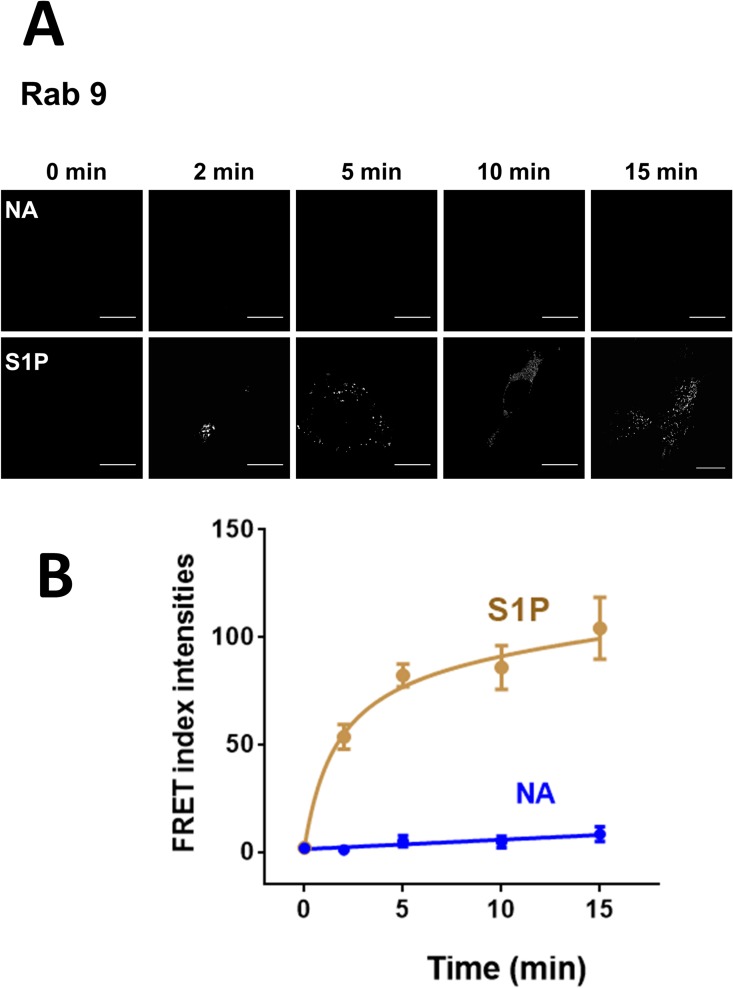
Time-course of the α_1B_-adrenergic receptor-Rab 9 interaction. Panel A: FRET index images of cells coexpressing Ds-Red-tagged α_1B_-adrenergic receptors and EGFP-tagged Rab 9 treated for the times indicated with 10 μM noradrenaline (NA) or sphingosine 1-phosphate (S1P). Scale bars: 15 μm. Panel B: Quantitative analysis of the data presented in Panel A. Plotted are the means and vertical lines representing the S.E.M of 5–7 experiments using different cell preparations. Noradrenaline, blue line and symbols; sphingosine 1-phosphate, brown line and symbols.

As expected, when the dominant negative mutant of Rab 9 (Rab 9-GDP) was employed we were unable to detect FRET under any of the conditions studied (Fig. 8, panel B). To our surprise in cells coexpressing α_1B_-adrenergic receptors and the Rab 9 constitutively active mutant (Rab 9-GTP), some signal in the “FRET channel” was observed emitting from the red fluorescent protein, DsRed, under baseline conditions, i. e., in the absence of added stimulus, although the increase was variable and it was not statistically significant (Fig. 8, panel C). Such baseline FRET was further increased by both noradrenaline and sphingosine 1-phosphate (Fig. 8, panel C). The FRET index analysis is presented in Fig. 8 (panel D).The time-course of the effects of noradrenaline and sphingosine 1-phosphate on FRET index was studied using cells coexpressing α_1B_-adrenergic receptors and either Rab 5 (Fig. 10) or Rab 9 (Fig. 11). It can be observed that noradrenaline induced a marked progressive increase in FRET in cell expressing Rab 5 but hardly had any effect on cells expressing Rab 9 (Figs. 10 and 11). In contrast, sphingosine 1-phosphate induced a small rapid increase in FRET (2 min) in cells expressing Rab 5, which vanished at later times (Fig. 10) and a rapid and progressive increase in FRET in cells expressing Rab 9 (Fig. 11).

**Fig 12:**
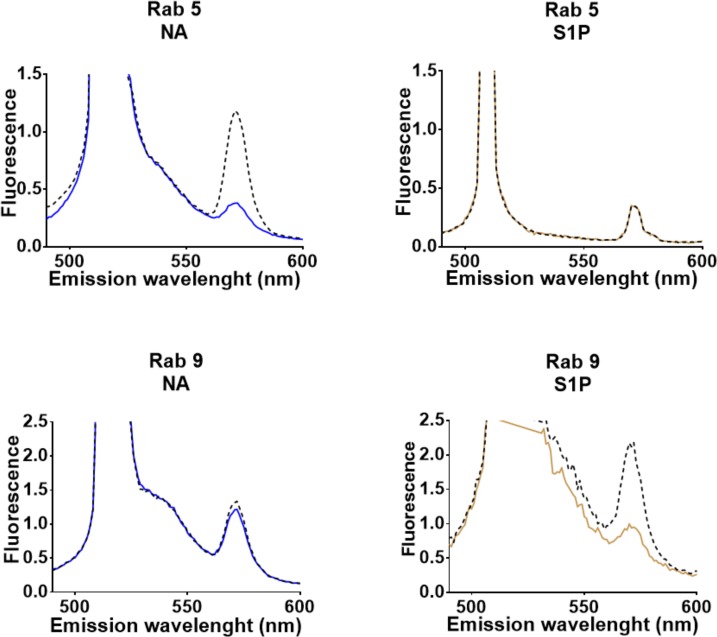
Representative emission spectra obtained from cells coexpressing Ds-Red-tagged α1B-adrenergic receptors and EGFP-tagged Rab proteins 5 or 9. The emission spectrum was captured at time zero (baseline, continuous lines), then cells were incubated for 15 min with 10μM noradrenaline (NA, blue lines) or with 1 μM sphingosine 1-phosphate (S1P, brown lines) and then, the emission spectrum was captured again (black dotted lines).

In order to confirm the previous data FRET was analyzed spectrofluorometrically using cell suspensions of cells cotransfected with the DsRed-tagged α_1B_-adrenergic receptors and the EGFP-tagged Rab proteins, Rab 5 or Rab 9. Samples were excited at 488 nm and fluorescence emission at 490–660 was scanned. Cells were incubated for 15 min in the absence of any stimulus or in the presence of 10 μM noradrenaline or 1 μM sphingosine 1-phosphate. Immediately after this incubation samples were excited again and fluorescence emission scanned. As illustrated in Fig. 12 in the absence of stimulus (solid lines) two peaks of fluorescence were observed, one of them, very large, in the wavelength 500–550 and another, smaller, in the range 550–575 nm. The first peak corresponded to the expected fluorescence of the EGFP whereas the second one corresponded to that of DsRed. It can be observed that treatment with noradrenaline markedly increased the FRET signal in cells transfected with Rab 5 but essentially no change was observed in cells transfected with Rab 9. However, an opposite effect was observed with the sphingosine 1-phosphate treatment, i. e., no change was observed in cells cotransfected with Rab 5 and a marked effect was observed in cells expressing Rab 9. No significant change was detected in EGFP fluorescence during the treatments. The data are consistent with what was observed using confocal microscopy.

**Fig 13:**
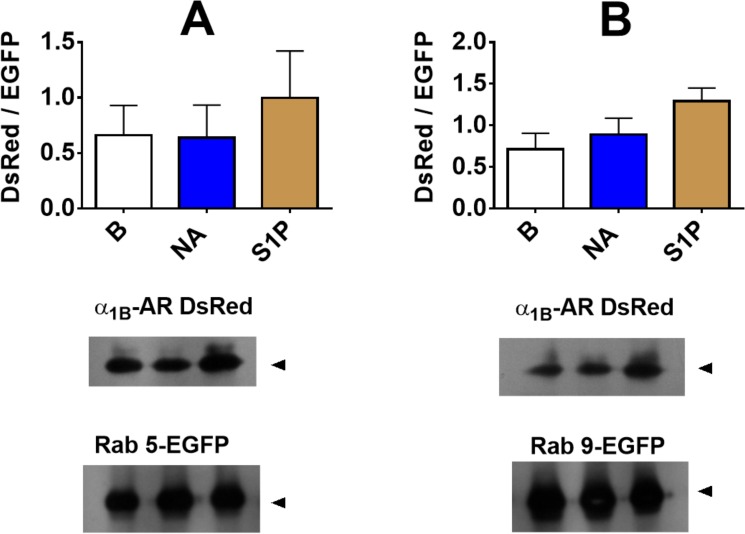
Coinmunoprecipitation of α_1B_-adrenergic receptors and the marker proteins. Rab 5 (panel A) and Rab 9 (panel B) were studied. Cells were incubated for 15 min in the absence of any agent or presence of 10 μM noradrenaline plus 1 μM propranolol (NA) or 1 μM sphingosine 1-phosphate (S1P). Cell lysates were obtained and the proteins were inmmunoprecipitated using an anti-EGFP antibody. The resulting immunocomplexes were analyzed by Western blots. The signal ratio (DsRed / EGFP) is plotted in the upper part of the panels; plotted are the means with vertical lines representing the S.E.M. of 3 experiments performed using different cell preparations. Representative blots are presented.

Coimmunoprecipitation experiments were performed Bands obtained in the Western blotting experiments were only detected when the tagged proteins were expressed. As shown in Fig. 7, α_1B_-adrenergic receptors coimmunoprecipitate with both Rab proteins (Rab 5, panel A; and Rab 9, panel B). Such coimmunoprecipitation was not modified significantly by any of the treatments (Fig. 13).

## Discussion

Our findings indicate that DsRed-tagged α_1B_-adrenergic receptors are functional and modulated by noradrenaline, sphingosine 1-phosphate and by pharmacological activation of protein kinase C (phorbol myristate acetate). These receptors are phosphoproteins whose phosphorylation state was increased in response to the above mentioned stimuli, which also triggered their internalization, as previously shown using wild type receptors and other constructions [42, 48, 57, 59, 71]. DsRed-tagged α_1B_-adrenergic receptors internalize in response to noradrenaline or sphingosine 1-phosphate, and colocalized with β-arrestin 2, i. e., these and β-arrestin 2 fell within the optical discrimination range (≤ 200 nm). Therefore, they behaved in a way indistinguishable from the wild type receptors. However, we cannot discard the possibility that the presence of the tag could alter their interaction with other cell components.

We previously showed that insulin growth factor I induces α_1B_-adrenergic receptor internalization, an effect that was blocked by hypertonic sucrose and Concanavalin A [48]. Hypertonic sucrose is known to inhibit receptor-mediated endocytosis by interfering with clathrin-coated pit formation [72]. Therefore, the data suggest that internalization associated with heterologous desensitization involves clathrin. Later work demonstrated that this insulin growth factor-induced effect involves the generation of sphingosine 1-phosphate and activation of the S1P_1_ receptor [42]. This α_1B_-adrenergic receptor internalization associated with heterologous desensitization markedly differs with that induced by noradrenaline. Sphingosine 1-phosphate-induced α_1B_-adrenergic receptor internalization was very intense with the formation of “lanes" or "rails" of receptors, migrating to intracellular / perinuclear regions of the cells which suggested interaction with cytoskeletal elements [43]. Other groups have shown that the G protein-coupled estrogen receptor 1 (GPR30), transits intracellularly associated with cytokeratin filaments [73] and that the transport of the α_2B_-adrenergic receptor from the endoplasmic reticulum to the cell surface, is controlled through its association with tubulin [74].

Agonist-induced endocytosis of GPCRs through the vesicular pathway has been studied in some detail; much less is known concerning receptor internalization associated with heterologous desensitization. The interaction of the receptor’s intracellular domains with specific targeting proteins appears to be crucial for their sorting into the different endosomal pathways [24]. Some receptors, such as the β_2_-adrenergic receptors, are internalized in response to agonist stimulation and recycle back to the plasma membrane very rapidly [75], whereas other receptors, such as the angiotensin II AT_1_ receptor, effect this at a much slower pace [63]. Common steps in intracellular trafficking pathways include invaginations of membrane for the formation of vesicles, transport to particular destinations and finally coupling and fusion with the target organelle. There is evidence that the majority of Rab proteins regulate the processes of focus/ coupling/ fusion. Mutants of Rab proteins are often characterized by inducing massive accumulation of particular types of vesicles. Therefore, the different members of the Rab family of GTPases are considered endosome markers, due to their restricted distribution and action. Although the regulatory components of the endocytic pathway are freely distributed in the cellular compartments, different combinations appear to be unique for each particular endosome class [15].

**Fig 14:**
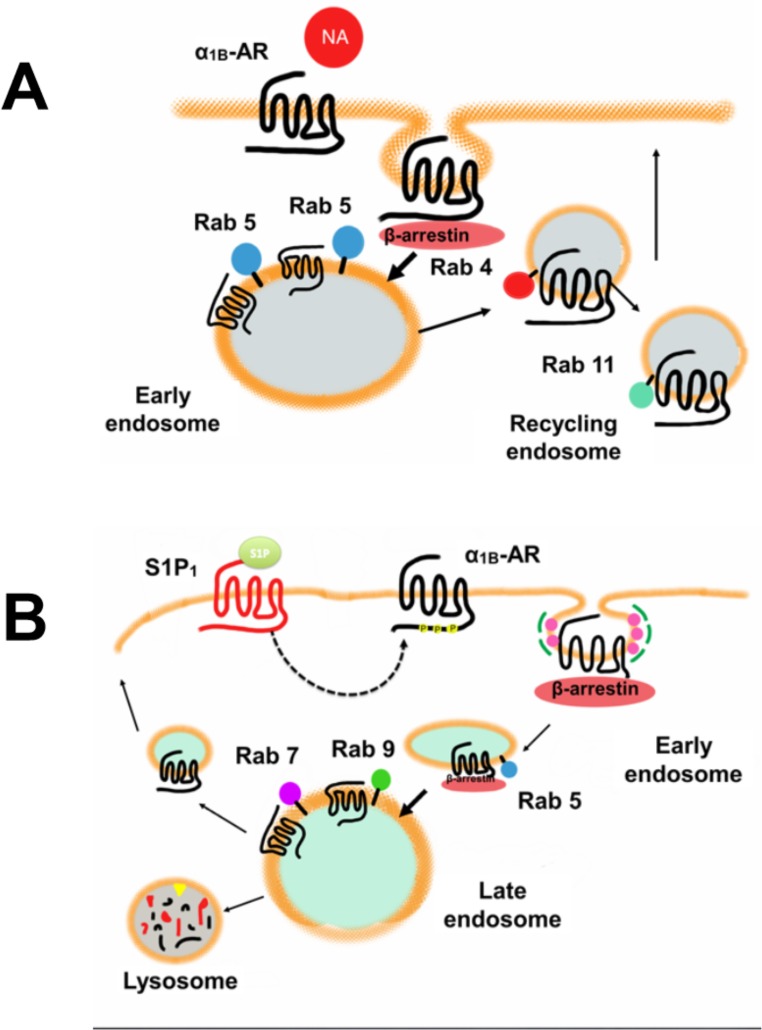
Models for α_1B_-adrenergic-Rab protein interaction. α_1B_-Adrenergic internalization and recycling back to the plasma membrane during homologous (panel A) and heterologous (panel B) desensitization is depicted. NA, noradrenaline; α_1B_-AR, α_1B_-adrenergic receptor; S1P, sphingosine 1-phosphate; S1P_1_, sphingosine 1-phosphate receptor type 1. Rab proteins are shown as membrane anchored proteins.

The major finding of this work is that α_1B_-adrenergic receptors interact differentially with subtypes of Rab proteins during homologous and heterologous desensitization. This was consistently observed using two approaches, profiting from FRET: scanning fluorescence in cell suspensions and sensitized-emission method employing cell images obtained with a confocal microscope. Agonist-activation targets these receptors to early endosomes and fast recycling back to the plasma membrane, as evidenced by their interaction with the early endosomes antigen 1, Rab 5, Rab 4 and Rab 11. Rab 5 is likely involved in initial endocytosis and in interaction with early endosomes [17], whereas Rab 4, appears to participate in the recycling of receptors back to the plasma membrane [24]. The GTPase activity of Rab 5 appears to be of great importance in the adrenoceptor-Rab interaction, because in cells expressing the dominant-negative mutant of this Rab, no FRET was observed. This is depicted in the model in Fig. 14 (panel A). Data on other receptors are consistent with this suggestion. On the one hand, some GPCRs (β_2_-adrenergic receptors, dopamine D2 receptors, and M4 muscarinic acetyl-choline receptors) have been shown to traffic to early endosomes, colocalizing with Rab 5 after its internalization in response to agonist stimulation. Rab 5 also controls the internalization of both endothelin receptors, A and B [18, 76–78]. The expression of a Rab 5 mutant (Rab 5-S34N) blocks the internalization of some GPCRs [18]. On the other hand Rab 4 regulates the intracellular sorting and distribution of transferrin receptors, low-density lipoprotein receptors, and epidermal growth factor receptors [33, 79] and controls the rapid recycling of proteins to the cell membrane [24]. Rab 5 is also implicated in Cannabinoid receptor 2 (CB2) internalization, but for recycling of this receptor Rab 11 and Rab 4 appear to be involved [80]. It is likely that Rab 4 and Rab 5 might act together to control input and output flows in the early endosomes, respectively, because these two proteins exhibit binding to the same effectors [24, 81]. Rab 11 participates in slow recycling of M4 muscarinic acetylcholine receptors [34] transferrin, calcium sensing receptor [82] and Angiotensin II receptors [83]. Our data also suggest a role for Rab 11 in the recycling of α_1B_-adrenergic receptors to the plasma membrane under heterologous desensitization (Fig. 13, panel A).

Proteins targeted for degradation are delivered to late endosomes and lysosomes. The boundary between late endosomes and lysosomes is difficult to determine, because both might contain lysosomal enzymes, their pH is acid (≈ 5.5) and their membranes have a similar composition. In some cases lysosomes can be identified based on their physical properties and due to the fact that they lack some of the proteins that are found in late endosomes [24, 84]. Rab 7 and Rab 9 are characteristic proteins of late endosomes, and receptor association with these organelles is strongly inhibited by the expression of a dominant-negative Rab 7 mutant, indicating that Rab 7 is essential for transport to late endosomes. However, it remains unclear whether Rab 7 is also necessary for transport to lysosomes [30]. Our data also showed that α_1B_-adrenergic receptors interact rapidly, but transiently, with Rab 5, and in a more sustained fashion with the marker proteins of the late endosomes, Rab 7 and Rab 9, when they are desensitized by sphingosine 1-phosphate (heterologous desensitization). This is depicted in Fig. 14 (panel B). These findings suggest that when α_1B_-adrenergic receptors are desensitized in a heterologous fashion and internalized, some of them recycle slowly, back to the plasma membrane and other receptors could be targeted to degradation. Rab 9 activity also appears to be essential for interaction with α_1B_-adrenergic receptors as evidenced by the absence of FRET when the dominant-negative mutant of this Rab was expressed. We were surprised by the fact that some FRET was detected under baseline conditions and it further increased when cells were treated with noradrenaline or sphingosine 1-phosphate when the constitutively active mutant of Rab 9 was expressed. One possibility is that this GTPase could be rate-limiting for slow recycling and degradation and that the constitutively active mutant might drive the basal receptor flux to these pathways. The clear effect of noradrenaline on FRET under these conditions suggests that some agonist-stimulated α_1B_-adrenergic receptors might use the slow recycling and degradation pathway through Rab 9 and that this likely could have been amplified once the limiting step was eliminated.

Coimmunoprecipitation studies allowed us to observe the interaction between Rab proteins and the adrenergic receptor. Such interaction could be direct or through other cell components. However, this methodology did not allow us to observe any change due to cell stimulation, as observed using the FRET index. Obviously, these methodologies explore different aspects. To observe FRET, a distance of 10 nm (100 Å) or less is required; this is, therefore, a strong indication of close proximity, whereas coimmunoprecipitation might reflect indirect association within large intracellular complexes. It is noteworthy that in the initial exploration using continuous recording in the “FRET channel” we observed that when cells are stimulated, the signal does not take place in an “off-on” manner but rather occurs in intermittent flashes within the cells (S1 Video); this signaling pattern is consistent with the idea that receptor-Rab complexes are formed and dissociate in a very dynamic fashion.

It has been reported that differences in the signaling of α_1A_- and α_1B_- adrenergic receptors could be due to different endosomal targeting [85]. α_1A_-Adrenergic receptors, when located in the plasma membrane, signal through calcium and ERK1/2 pathways but, when translocated to deeper endosomes, continue signaling through ERK1/2 and also activate the p38 pathway. α_1B_-Adrenergic receptors signal through calcium and ERK1/2 only when located in the membrane, and the signals disappear after endocytosis or by disruption of the membrane lipid rafts by methyl-β-cyclodextrin [85]. Our results indicate that α_1B_-adrenergic receptors can be internalized and that they target different types of endosomes when desensitized through homologous or heterologous processes, it is currently unknown whether these differences entail any functional significance in postendocytic signaling. How cells “sense” that the receptors have been desensitized in one way or another is currently unknown. However, α_1B_- adrenergic receptors are phosphorylated at different sites by G protein-coupled receptor kinases and protein kinase C [50]. It is, therefore, possible that the differences in the sites phosphorylated in these processes might determine their internalization and trafficking.

In conclusion, our data indicate that α_1B_-adrenergic receptors are recruited to early endosomes when desensitized by agonist exposure (homologous), while when desensitized by other stimuli (heterologous) they are initially but transiently recruited to early endosomes and then transferred to late endosomes. Intracellular trafficking of GPCRs regulates receptor availability at the plasma membrane and in this manner modulates the cellular response to agonists. Rab GTPase proteins regulate the traffic of membrane vesicles, and identification of the networks that control GPCR trafficking is essential for understanding these receptors signaling.

## Supporting Information

S1 Video α_1B_-Adrenergic-Rab 5 protein interaction (evidenced by “flashes”); continuous recording in the FRET channel during homologous desensitization. (AVI)
